# Physician education on World Asthma Day aids in disease management during the COVID-19

**DOI:** 10.1186/s13223-022-00741-8

**Published:** 2022-12-30

**Authors:** Angyang Cao, Yanling Zhou, Wenjun Luo, Dan Lv, Zhonghao Shao, Binbin Zhu, Jianhua Wang

**Affiliations:** 1 Ningbo University School of Medicine, Ningbo, China; 2 The Affiliated Hospital of Medical School of Ningbo University, Ningbo, China; 3 Affiliated Hospital of Shaoxing University, Shaoxing, China

**Keywords:** Mental health, Psychological problem, Asthma, Public education

## Abstract

Anxiety and depression can negatively affect the management of asthma. The study aimed to assess the psychosocial effects of asthma patients during COVID-19 and analyze potential risk factors and interventions.

In June 2022, the “Questionnaire Star” electronic questionnaire system was used to collect data. A total of 98 asthma patients from the affiliated hospital of the medical school of Ningbo University were invited to complete the questionnaires. According to our study, the prevalence of symptoms of anxiety and depression in the asthma patients in the institution was 91.8 and 77.6%, respectively. Patients who had an asthma exacerbation in the previous two months were more likely to have anxiety symptoms (OR = 0.142 95%CI 0.025–0.820), while patients who did not participate in asthma day activities were more likely to have anxiety symptoms than those who did (OR = 0.130 95%CI 0.022–0.762).

This study found that routine disease educational lectures on asthma day can successfully alleviate asthma sufferers' anxiety and depression.

## Introduction

In late March 2022, when the Omicron strain of COVID-19 hit Shanghai, the local government began two-month silent management (from 1 April to 31 May 2022),which included closing public places, canceling large events, and discouraging crowds from gathering [1]. As a city in the Yangtze River Delta region that has close exchanges with Shanghai, Ningbo has to implement similar restrictive lockdowns, causing huge inconvenience to people's lives and jobs.

The capacity of Ningbo patients with asthma to visit their physicians or general practitioners has been seriously affected. It has been reported that anxiety and depression have increased significantly during the new coronavirus pandemic [2, 3], meanwhile, anxiety and depression may associate with poorer control and quality of life in adults with asthma [4]. We wonder about the quality of self-management of asthma patients in Ningbo during the Shanghai epidemic, so we launched a series of online and offline educational lectures on asthma management in early May, in response to the theme of this year's “World Asthma Day” “Bridging the Gap in Asthma Care”, raising public awareness of asthma, and popularizing asthma knowledge of prevention and treatment, and promoting the standardized treatment of asthma. A cross-sectional survey was conducted to understand patients' mood and quality of life or management of the disease during the period of lockdown retrospectively in June.

## Research objects and methods

### From June 7th to June 30th in 2022

We distributed online questionnaires to 120 asthma patients in the outpatient department. Subsequently, we received 98 responses accordingly with an effective recovery rate of 81.6%. At the beginning of the questionnaire, we informed participants that they would sign the consent by default if they completed the survey. All of the patients were invited to voluntarily participate in the online survey. Ethics approval was obtained from the Clinical Ethics Committee of the Affiliated Hospital of the medical school of Ningbo University.

### Survey method

We used “Questionnaire Star” to collect data as previously described [5]. The content of the questionnaire includes general information, asthma drug acquisition, and other issues. All questionnaires are anonymous.

### Survey scale

#### Anxiety symptoms

We employed the Chinese version of GAD-7 to assess the anxiety symptoms of patients [6]. The GAD-7 is a self-report questionnaire that screens and measures the severity of generalized anxiety disorder. Participants rated 7 items on a 4-point scale from 0 (not at all) to 3 (almost every day) based on symptom frequency over the past two weeks. The total score ranged from 0 to 21, with higher scores indicating more severe anxiety symptoms, 0–4 points no anxiety disorder, 5–9 points may have mild anxiety disorder, 10–13 points may have moderate anxiety disorder, 14–18 points may have moderate to severe anxiety disorder, 19–21 may have severe anxiety disorder [7]. In China, GAD-7 has been widely used and confirmed the good reliability and validity of GAD-7[8]. The presence of mild anxiety symptoms was defined as a total score of ≥ 5 points in the GAD-7 in this survey.

#### Depressive symptoms

Patients' depressive symptoms were assessed using the PHQ-9[9], a 9-item self-report measure of depression severity, in which participants rated each item on a 4-point scale based on the frequency of symptoms over the past 2 weeks, ranging from 0 (not at all) to 3 (almost every day) on a scale ranging from 0 to 27 on a scale of 0–4 with no depression, 5–9 with mild depression, 10–14 with severe depression, 15–19 with possible Moderate to severe depression, 20–27 may have severe depression. PHQ-9 has been widely used in China, and proved that PHQ-9 has good reliability and validity [10]. The mild depressive symptom was defined as a total score of ≥ 5points in the PHQ-9 in this survey.

#### Patient statistics

We designed the characteristics of the participants on the questionnaire, including gender, age, weight, smoking history, occupational status, and asthma history.

### Statistical methods

Categorical variable statistics were used in this statistical analysis. The categorical variables were reported as percentages, and the chi-square test was used to determine statistical significance. To evaluate the results of the chi-square test p 0.05 in categorical variables, the binary logistic regression analysis was performed. The Hosmer–Lemeshow goodness-of-fit statistic was used to assess model discrimination and calibration. P = 0.05 on both sides was judged statistically significant. To acquire statistical data, these statistical instruments included SPSS v25.0 (IBM) and "questionnaire star."

## Results

### Basic information

A total of 98 patients were investigated, including 52 males (53.06%) and 46 females (46.94%); 0 (0%) under the age of 18, and 4 (4.08%) between 18 and 25 years old. 20 (20.41%) aged 26–30; 28 (28.57%) aged 31–40, 23 (23.47%) aged 41–50, 18 (18.37%) aged 51–65; 5 aged over 65 (5.1%); 36 (36.73%) weighed 50–60 kg, 23 (23.46%) weighed 61-70 kg, 26 (26.53%) weighed 71–80 kg, 12 (12.24%) weighed 81-90 kg, 1 (1.02%) weighed 100 kg;7(7.14%) are students,5(5.10%) are in sales,7(7.14%) are public relations personnel,1(1.02%) is in the service industry,5(5.1%) are administrative staff, 20 (20.4%)are in professional jobs (doctors, teachers, lawyers, accountants, etc.),53(54.08%) 53(54.08%) are retirees or farmers; 24 (24.48%)had a smoking history, 74 (75.52%) had no smoking history, the 24 patients with a history of smoking were in the age group over 26 years, mainly in the 31–65 years age group; 36(36.73%) had an acute asthma attack in the last two months, and 62 (63.27%) had no attack, the 36 patients with acute attack of asthma ranged in age from 18 to 65 years, mainly in the 26–65 years age group.

### Mental health status

**Fig. 1:**
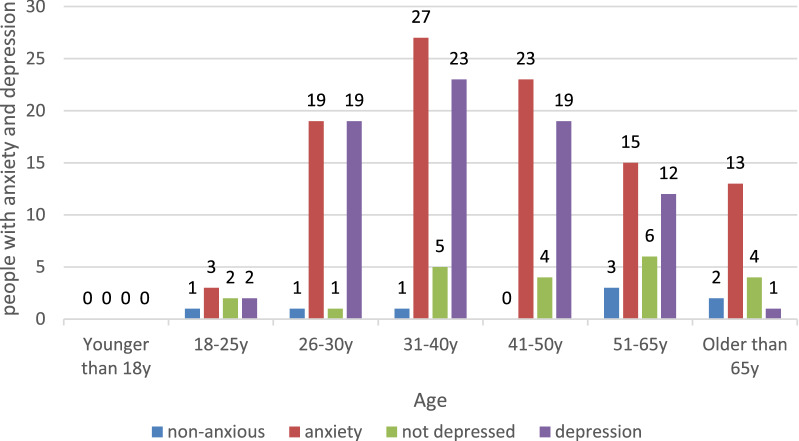
Age distribution of anxiety and depression symptoms

The questionnaire showed that 90 (91.8%) people in this survey had anxiety-related symptoms, and the questionnaire showed that 76 (77.6%) people in this survey had depression-related symptoms (Table 1). Patients with anxiety and depression also showed a different age distribution (Fig. 1).

**Table 1:** Sample characteristics and univariate analysis of variables related to anxiety and depressive symptoms

Variables	n (%)	Anxiety symptoms (GAD-7 score)		*p*	Depressive symptoms (PHQ-9 score)		*p*
		5 < (n = 8)	≥ 5(n = 90)		5 < (n = 22)	≥ 5(n = 76)	
Demographics							
Gender				0.469			0.630
Male	52(53.1)	3 (37.5)	49 (54.4)		52(53.1)	13 (59.1)	
Female	46(46.9)	5 (62.5)	41 (45.6)		46(46.9)	9 (40.9)	
Asthma control in last two months				0.048			0.209
Sudden attack	36(36.7)	6 (75.0)	30 (33.3)		11 (50.0)	25 (32.9)	
No sudden onset	62(63.3)	2 (25.0)	60 (66.6)		11 (50.0)	51 (48.1)	
Concern about Omicron on asthma				0.139			0.802
Yes	36(36.7)	5 (62.5)	31 (34.4)		9 (40.9)	27 (35.5)	
No	62(63.3)	3 (37.5)	59 (65.6)		13 (59.1)	49 (64.5)	
Expect the outbreak would cease immediately				1.000			1.000
Yes	97(98.98)	8 (100)	89 (98.9)		22 (100)	75 (98.7)	
No	1(1.02)	0 (0)	1 (1.1)		0 (0)	1 (1.3)	
Get instructions from the internet				0.139			0.000
Yes	17 (17.35)	3 (37.5)	14 (15.6)		10 (45.5)	7 (9.2)	
No	81(82.65)	5 (62.5)	76 (84.4)		12 (54.5)	69 (90.8)	
Difficulty to acquire medicine				0.188			0.225
Yes	10 (10.2)	2 (25.0)	8 (8.9)		4 (18.2)	6 (7.9)	
No	88 (89.8)	6 (75.0)	82 (91.1)		18 (81.8)	70 (92.1)	
Total isolated from the society				0.157			0.049
Yes	2 (2.0)	1 (12.5)	1 (1.1)		2 (9.1)	0 (0)	
No	96 (98.0)	7 (87.5)	89 (98.9)		20 (90.9)	76 (100)	
Impact of nucleic acid test on transportation and hospital consultation				0.727			0.091
Yes	55 (56.12)	4 (50.0)	51 (56.7)		16 (72.7)	39 (51.3)	
No	43 (43.88)	4 (50.0)	39 (43.3)		6 (27.3)	37 (48.7)	
Participate in the world asthma day education program				0.044			0.000
Yes	11 (11.22)	3 (37.5)	8 (8.9)		8 (36.4)	3 (3.9)	
No	87 (88.78)	8 (62.5)	82 (91.1)		14 (63.6)	73 (96.1)	
Disturbed self-management of asthma				0.050			0.000
Yes	37 (37.76)	6 (75.0)	31 (34.4)		16 (72.7)	21 (27.6)	
No	61 (62.24)	2 (25.0)	59 (65.6)		6 (27.3)	55 (72.4)

**Fig. 2:**
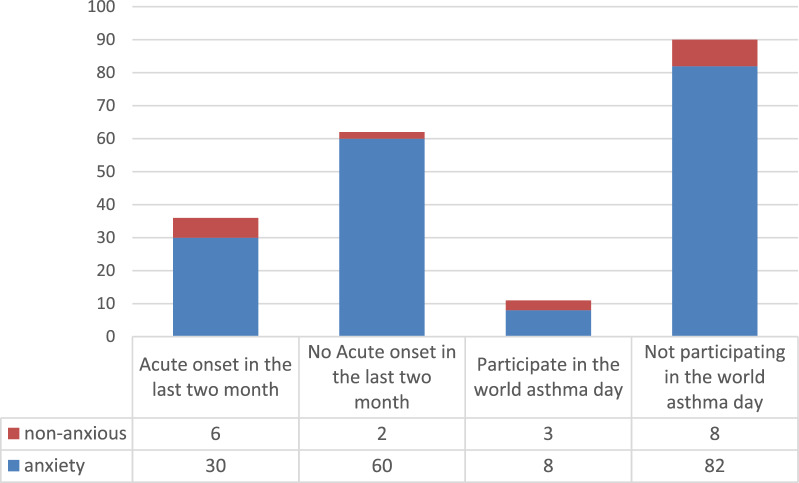
Variables related to anxiety symptoms

According to the data presented above, 91.8 percent and 77.6 percent of the patients, respectively, have symptoms of anxiety and depression, which is significantly higher than the general population in China, demonstrating that asthma patients face greater 25psychological stress as a result of the epidemic. Patients who had an asthma exacerbation in the previous two months were more likely to have anxiety symptoms (OR = 0.142 95 percent range 0.025–0.820), while patients who did not participate in asthma day activities were more likely to have anxiety symptoms than those who did (OR = 0.130 95 percent range 0.022–0.762) (Fig. 2) (Tables 2 and 3).

**Table 2:** Multivariate logistic regression analysis of variables related to anxiety symptoms

Anxiety symptoms(GAD-7 score ≥ 5)			
	*p*	OR	95% CI
Acute onset in the past two months	0.048	0.142	0.025–0.820
Participate in the world asthma day	0.044	0.130	0.022–0.762

**Table 3:** Multivariate logistic regression analysis of variables related to depressive symptoms

Depression symptoms(PHQ-9 score ≥ 5)			
	*p*	OR	95% CI
Get an education from the internet	0.000	0.220	0.060–0.802
Participate in the world asthma day	0.000	0.072	0.017–0.305
Disturbed self-management of asthma	0.000	0.274	0.082–0.919

**Fig. 3:**
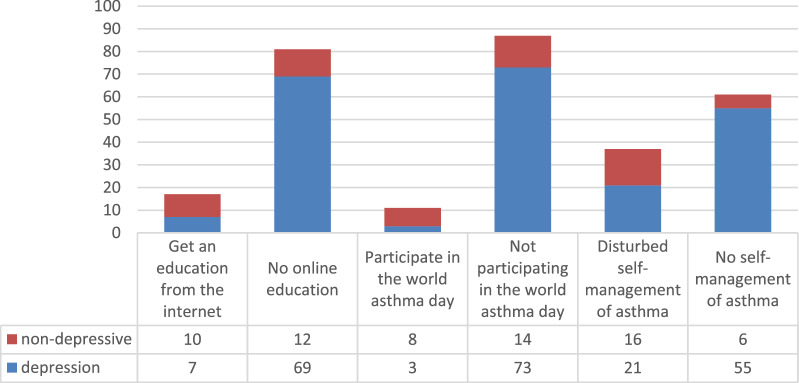
Variables related to depression symptoms

Patients who did not participate in education were more likely to experience depression than those who participated in remote instruction from the internet (OR = 0.220 95% interval 0.060–0.802) and asthma day activities (OR = 0.072 95% interval0.017–0.305), and those who felt that they were Patients whose self-management was disrupted by the epidemic (OR = 0.274 95% interval 0.082–0.919) were more likely to have depressive symptoms (Fig. 3).

## Discussion

Asthma, as a chronic heterogeneous illness, has been linked to psychiatric symptoms and mental problems. There is substantial evidence that persons with asthma have a worse quality of life and a higher frequency of mental problems [11, 12]. Emotional illnesses, such as anxiety and sadness, are among the detrimental effects of asthma symptoms [13]. The pathophysiological factors behind anxiety and depression in asthma are many and diverse. Asthmatic patients live in continual anxiety of illness development and an attack at any time. Chronic psychological distress may create a pro-inflammatory state and be related with increased superoxide, cytokine, and leukocyte production [13]. Furthermore, neurokinins and P substance (byproducts of asthma pulmonary neurogenic inflammation) have a direct effect on the central nervous system, generating anxiety states [14]. In asthma patients, inhaled corticosteroids have been linked to depressive symptoms. Long-term corticosteroid usage has been linked to hypothalamic–pituitary–adrenal axis dysregulation [15]. Asthmatic patients are more fearful of contracting SARS-CoV-2 infection, especially during the COVID-19 pandemic. Asthmatic individuals are also more likely to suffer from anxiety and sadness [16]. Many studies show that psychological illnesses are also linked to worse asthma outcomes [17, 18]. Persistent asthma may be caused by a mental condition, which is connected with poor asthma control and reduced asthma-related quality of life [19].

Four essential components of asthma management include patient education, monitoring of symptoms, control of triggering factors, and pharmacologic therapy. Patient education on asthma decreases exacerbations and improves control, and is a necessary and critical component [20]. Asthma is a chronic disease that requires long-term management. At the most basic level, education includes information about asthma, its causes and treatments [21]. In the crisis of this epidemic, physicians may get stressed themselves, but for the well-being of patients, it is worthwhile to share knowledge through the internet or other media, as timely scientific popularization could reduce the number and frequency of acute attacks of the asthma patient. The doctors’ instructions include the following items: differences between relievers and controls, inhaler use, self-monitoring of symptoms, self-management plans (how to recognize and respond to worsening asthma), avoidance of environmental allergens and so on. The doctor's advice increased the patient's awareness of asthma and effectively reduced the frequency of attacks.

There was increased stress under lockdown, especially for patients with chronic diseases [1], and the incidence of anxiety and depression for asthma patients in our study was high, which is consistent with the findings of other studies [22, 23]. Previous studies in allergic patients indicate the frequency of anxiety due to the COVID-19 lockdown [24]. The study by Xu et al. of anxiety among patients with allergic rhinitis during the COVID-19 pandemic reported a significantly higher levels of anxiety [25]. Our study shows comparable results for anxiety scores in asthma patients, who often have allergies as well. Although there was some private confounder that we did not cover in the investigation, like income losses, or another big event in life (lost job, cancellation of the wedding ceremony, and so on), we found that physicians do reduce the degree of anxiety and depression of patients by strengthening the communication and offering disease education. In the crisis of this epidemic, physicians may get stressed themselves [26], but for the well-being of patients, it is worthwhile to share knowledge through the internet or other media, as timely scientific popularization could reduce the number and frequency of acute attacks of the asthma patient.

## Conclusion

The asthma patients of Ningbo experienced high levels of anxiety and depression during the shanghai epidemic, and doctors' sharing and interpretation of chronic disease self-management knowledge eased patients' stress and depression levels. During the pandemic, doctors have mainly used the Internet or other media to disseminate knowledge about asthma to the general public. At the same time, they also provide personalized treatment for patients offline and spiritual support for those with psychological disorders.Therefore, during the COVID-19 epidemic, doctors are encouraged to provide professional education by different means to help patients.

## Data Availability

If the request is reasonable, the original data can be requested from the corresponding author.
